# The identification of a blood circular RNA signature that differentiates Chikungunya virus infection

**DOI:** 10.3389/fgene.2025.1602177

**Published:** 2025-04-29

**Authors:** Xue Bai, Hanlin Yan, Guoxia Wen, Shaoxun Yuan, Wanjun Gu

**Affiliations:** ^1^ School of Artificial Intelligence and Information Technology, Nanjing University of Chinese Medicine, Nanjing, Jiangsu, China; ^2^ Liangzhu Laboratory, Zhejiang University School of Medicine, Hangzhou, Zhejiang, China; ^3^ Collaborative Innovation Center of Jiangsu Province of Cancer Prevention and Treatment of Chinese Medicine, Nanjing University of Chinese Medicine, Nanjing, Jiangsu, China; ^4^ Jiangsu Province Engineering Research Center of TCM Intelligence Health Service, Nanjing University of Chinese Medicine, Nanjing, Jiangsu, China

**Keywords:** Chikungunya virus, circular RNA, peripheral whole blood, diagnostic biomarker, infectious disease

## Abstract

**Objective:**

Chikungunya virus (CHIKV) is a re-emerging mosquito-borne human pathogen, which poses critical threats to the public health. However, an effective diagnostic method for early CHIKV infection remains scarce. Circular RNAs (CircRNAs) are a novel class of RNAs with important biological functions. They have been shown to be promising biomarkers for many human diseases. In this study, we sought to identify circRNA biomarkers in human peripheral whole blood for the early diagnosis of CHIKV infection.

**Design:**

Candidate circRNA biomarkers were identified by group comparison using a case-control study, which was further validated using an independent cohort. The performance of this signature and its correlation with clinical factors were estimated in both cohorts.

**Results:**

Using two public RNA-seq datasets of CHIKV infection, we developed and validated a 13-circRNA based blood signature that can discriminate CHIKV infectious patients from healthy controls. Furthermore, this blood circRNA signature was correlated with viral load in patients with CHIKV infection. Functional analysis implicated that these biomarker circRNAs were involved in the activation and regulation of immune processes against CHIKV infection.

**Conclusion:**

Collectively, our findings indicated that peripheral blood circRNAs were potential biomarkers for the early diagnosis of CHIKV infection.

## Introduction

Chikungunya virus (CHIKV) is an arthropod-borne virus transmitted to human mainly by infected *Aedes* mosquitoes in tropical and subtropical area (George et al., 2019). The CHIKV infection causes chikungunya fever, a disease typically accompanied by myalgia, fever, rashes or a classic symptom of arthralgia (Vairo et al., 2019). With the convenience and prevalence of global transportation, CHIKV has re-emerged and become prevalent throughout the world, posing growing threats to public health and economic growth (Mourad et al., 2022). Unfortunately, there is neither vaccine nor any appropriate antiviral therapy to treat CHIKV infection (Vairo et al., 2019). The current diagnostic methods for CHIKV infection are mainly using enzyme-linked immunoassay (ELISA) or reverse transcription-polymerase chain reaction (RT-PCR) (Mourad et al., 2022; Hakim and Aman, 2022). These two methods differ in sensitivity and specificity, but both require several days of viral infection before CHIKV-specific antibodies or nucleic acids can be detected in the sample (Mourad et al., 2022; Hakim and Aman, 2022). The delayed diagnosis of CHIKV infection is detrimental to the rapid control of its transmission. Thus, a quick and effective diagnostic tool is an urgent need for better prevention and management of the disease. Recent studies have elucidated the mechanisms by which CHIKV evades host immune responses, such as utilizing the *Mxra8* receptor for cellular entry (Zhang et al., 2018), suppressing interferon signaling pathways (Suzuki, 2023), and developing resistance to host restriction factors (Mounce et al., 2017), thereby underscoring the urgent need for novel diagnostic strategies capable of detecting infection at earlier stages with greater accuracy.

Circular RNAs (circRNAs) are a group of non-canonical RNAs with closed loop structure (Chen, 2020). Extensive studies have demonstrated that circRNAs have important biological functions in many physiological processes, and their aberrant expression is associated with many human diseases (Chen, 2020). Notably, circRNAs participate in host-pathogen interactions (Awan et al., 2021), with some interfering with proteins (Min et al., 2023) or modulating immune signaling pathways during infections (Yan and Chen, 2020). Due to their abundant, spatial-temporal specific expression (Zhou et al., 2018) and high stability (Wen and Gu, 2022), blood circRNAs have been shown to be promising non-invasive biomarkers for the diagnosis, prognosis, relapse monitoring and subtype stratification of human diseases (Wen et al., 2021a). Compared to conventional diagnostic methods such as RT-PCR and ELISA, which require 3–5 days post-infection for reliable detection, circRNAs provide a more stable and earlier biomarker alternative (Huits et al., 2018; Yap et al., 2010). In our previous analysis, we have identified a circRNA-based molecular signature from peripheral blood mononuclear cells (PBMCs) that could discriminate active TB patients from healthy controls with an area under the receiver operating characteristic curve (AUC) of 0.946 (Qian et al., 2018). Given the potential application of blood circRNAs in diagnosis of human infectious diseases, we hypothesize that a blood circRNA signature may be able to discriminate patients with CHIKV infection from healthy controls.

In this study, we sought to investigate the prospect of using peripheral blood circRNAs as diagnostic biomarkers for CHIKV infection. Firstly, we characterized the circRNA expression profiles in the whole blood samples of CHIKV patients. Next, we identified circRNA-based signatures that could differentiate CHIKV patients from healthy controls using differentially expressed circRNAs. Then, we validated the performance of this circRNA-based signature as well as its correlation with disease phenotypes in an independent cohort. Finally, we investigated the functional implications of this identified circRNA signature during CHIKV invasion.

## Materials and methods

### Overview of the study cohorts

**FIGURE 1:**
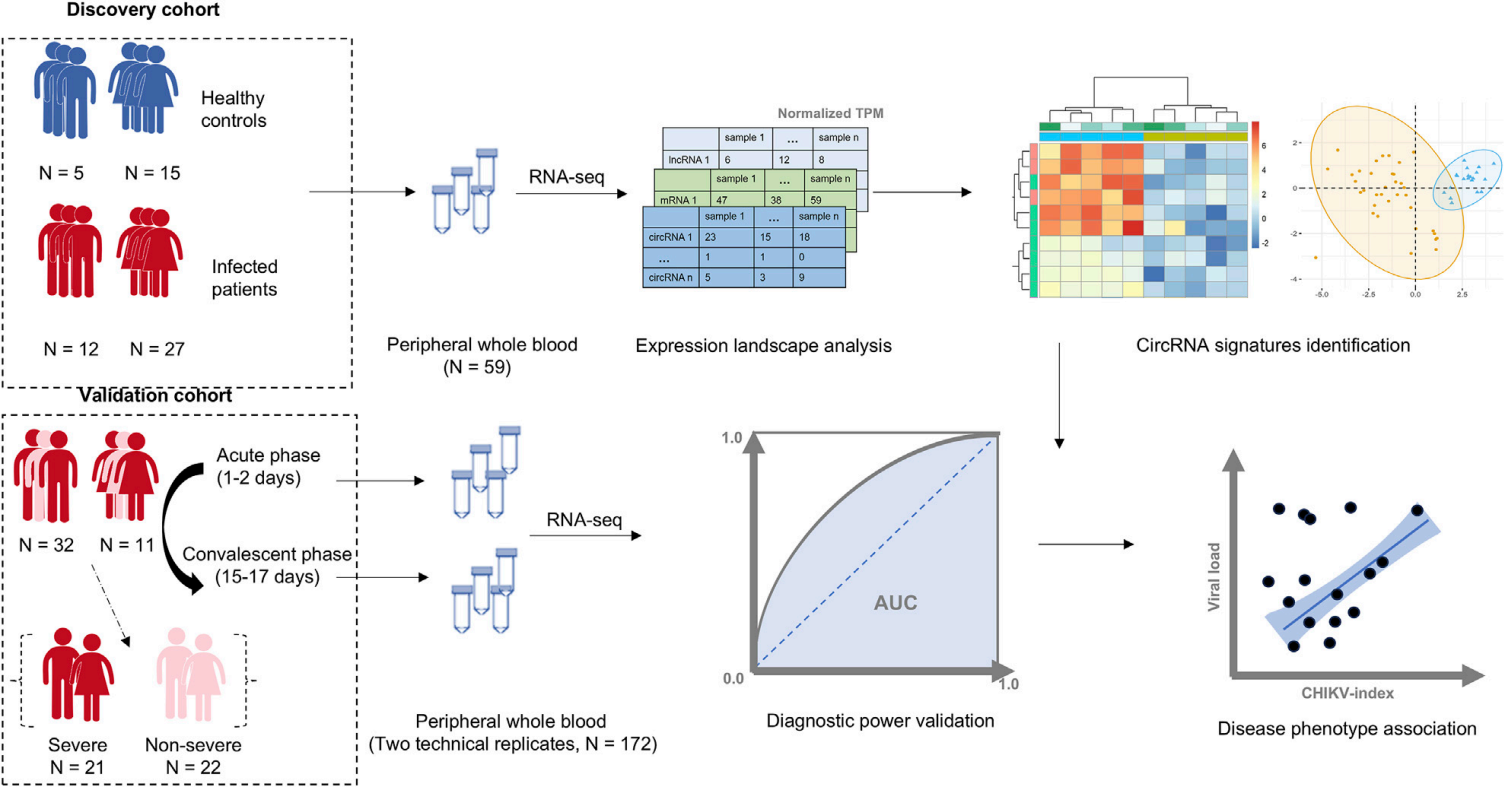
The workflow of this study. A cohort of 39 patients with Chikungunya virus (CHIKV) infection and 20 healthy controls were used as the discovery cohort. The whole transcriptome data from peripheral whole blood samples in this cohort was profiled, and a blood circRNA signature was identified to differentiate patients with CHIKV infection from healthy controls. The diagnostic power of the blood circRNA signatures was assessed in a validation cohort, which contained a total of 172 whole blood samples from acute (1–2 days post symptom onset) and convalescent phase (15–17 days post symptom onset) of CHIKV infection in 43 pediatric cases with two technical replicates. The correlation between the diagnostic score of blood circRNA signature and disease phenotypes, including viral load and disease severity, was also investigated.

We collected two total RNA-seq datasets of whole blood samples from patients with CHIKV infection from the European Nucleotide Archive (ENA) (Yuan et al., 2023) (Figure 1; Supplementary Table S1). To develop a circRNA signature for CHIKV infection diagnosis, we separated these two datasets into a discovery dataset and an independent validation dataset (Figure 1). For the discovery dataset, RNA-seq data of 59 whole blood samples were downloaded from ENA (accession number PRJNA507472), including samples from 39 patients with CHIKV acute infection and 20 healthy controls (Soares-Schanoski et al., 2019). For the validation dataset, a total of 172 whole blood RNA-seq data were downloaded from ENA (accession number PRJNA390289) as well. This dataset includes samples from acute (1–2 days post symptom onset) and convalescent phase (15–17 days post symptom onset) of CHIKV infection in 43 pediatric patients (Michlmayr et al., 2018). Moreover, these patients were grouped into 21 severe CHIKV infectious types and 22 less-severe CHIKV infectious types based on their clinical symptoms (severe type: peak temperature >38.5°C or a nadir platelet count <100,000 mm^-3^) (Michlmayr et al., 2018).

### Expression quantification of blood RNA transcripts

For each RNA-seq dataset, we identified the expressed circRNA transcripts using *CIRI-full* (Zheng et al., 2019) with *GRCh38* reference genome, *Ensembl* 94 gene annotation and the default parameters. Next, we constructed a reference library of expressed blood circular transcripts by combining the *de novo* constructed circular transcripts in annotated human genes from *CIRI-full* output and the known blood circRNA transcripts from *isoCirc* catalog (Xin et al., 2021). Then, we quantified the expression values of both circular and linear RNA transcripts using *AQUARIUM* (Wen et al., 2021b) with the compiled reference library of circular transcripts, *Ensembl* 94 gene annotation and the default parameters. After calculating the transcripts per million (TPM) values for all linear and circular RNA transcripts in each RNA-seq dataset, we integrated all the expressed transcripts in the discovery or validation datasets. Finally, those lowly expressed transcripts (a transcript that has a TPM value smaller than one in more than 25% samples) were excluded for further analysis. For circRNAs, the transcripts whose parental genes were not protein coding genes or lncRNAs were further filtered out.

### Differential expression analysis

To investigate the transcriptome changes between CHIKV patients and healthy controls or between acute-phase and convalescent-phase samples, we imported the transcript expression profiles from *AQUARIUM* output using *tximport* (Soneson et al., 2015) and calculated the expression differences of both circular and linear RNA transcripts by performing a linear model (*lm* function) in *R* platform with controlling potential confounding factors of age and gender as follows:

$$lm\;\left(Expression\;\left(\text{TPM}\right)\;∼\;Diagnosis+Gender+Age\right)$$

To construct the circRNA signature that differentiates CHIKV patients from healthy controls, we identified all circular transcripts with |log_2_(fold change)| larger than 0.5 and adjusted *P*-value less than 0.05 in differential expression analysis between the patients with CHIKV infection and healthy controls in the discovery cohort. These thresholds were chosen to ensure the identification of circRNAs with biologically significant expression changes while maintaining statistical rigor. Specifically, the selection of |log2(fold change)| > 0.5 is based on the need to identify biologically meaningful differential expressions while minimizing noise or insignificant variations. This threshold has been shown effective in previous infectious disease studies investigating circRNA biomarkers (Yao et al., 2021). Similarly, the adjusted *P*-value threshold of less than 0.05 ensures statistical robustness by controlling for false-positive rates, particularly in large-scale transcriptomic analyses. Together, these criteria were designed to capture circRNAs with significant diagnostic potential while maintaining rigor and reliability.

### Predicative power estimation of circRNA signatures

We applied a scoring scheme used in our previous studies to assign a circRNA-based CHIKV-index for each human subject (Qian et al., 2018; Gu et al., 2020). The circRNA-based CHIKV-index is calculated as the formula below.

$$I=\sum_{i=1}^{n}w_{i}\left(e_{i}-u_{i}\right)∕\tau_{i}$$

Here, *I* was the circRNA-based CHIKV-index; $w_{i}$ was the weight of circRNA *i*. $w_{i}$ = 1 if circRNA *i* was upregulated in the case group, while $w_{i}$ = −1 if circRNA *i* was downregulated in the case groups. $e_{i}$ was the expression level of circRNA *i*. $u_{i}$ and $\tau_{i}$ were the mean and standard deviation of the expression values of circRNA *i* across all the samples, respectively. A higher CHIKV-index implies a higher likelihood of CHIKV infection.

### Functional enrichment analysis

To explore the biological function of CHIKV infection related circular RNA transcripts, we performed the Gene Set Enrichment Analysis (GSEA) on circRNA expressions of blood samples in patients with CHIKV infection and healthy controls in the discovery cohort using *gseGO()* function (Subramanian et al., 2005), and visualized the enriched gene sets using *enrichplot()* function in *clusterProfiler* package (Yu et al., 2012). Biological pathways (BP) with *P*-value less than 0.05 were considered as significantly enriched.

### Statistical analysis

All statistical analyses were performed using the *R* platform. The *Kolmogorov-Smirnov* (*KS*) test was computed for comparison of the cumulative distribution of TPM values of circRNAs between patients with CHIKV infection and healthy controls in the discovery dataset by using the “*ks.test*” function. Student’s t*-*test was performed for groupwise comparisons of circRNA signatures in the discovery dataset by using the “*t.test*” function, while paired *t*-test was used for the comparisons in the validation dataset. If multiple testing should be accounted for, the *Benjamini–Hochberg* procedure was applied for *P*-value correction using the “*p.adjust*” function. Principal component analysis (PCA) of the circRNA signatures was performed using the “*fviz_pca_ind*” function within the package “factoextra”. The *AUC* was computed using the “*roc*” function within the package “*pROC*”.

### Data availability

The raw sequencing data and the associated sample metadata used in this study were accessible from the ENA database (Yuan et al., 2023).

## Results

### The circRNA expression landscape in human whole blood samples

**FIGURE 2:**
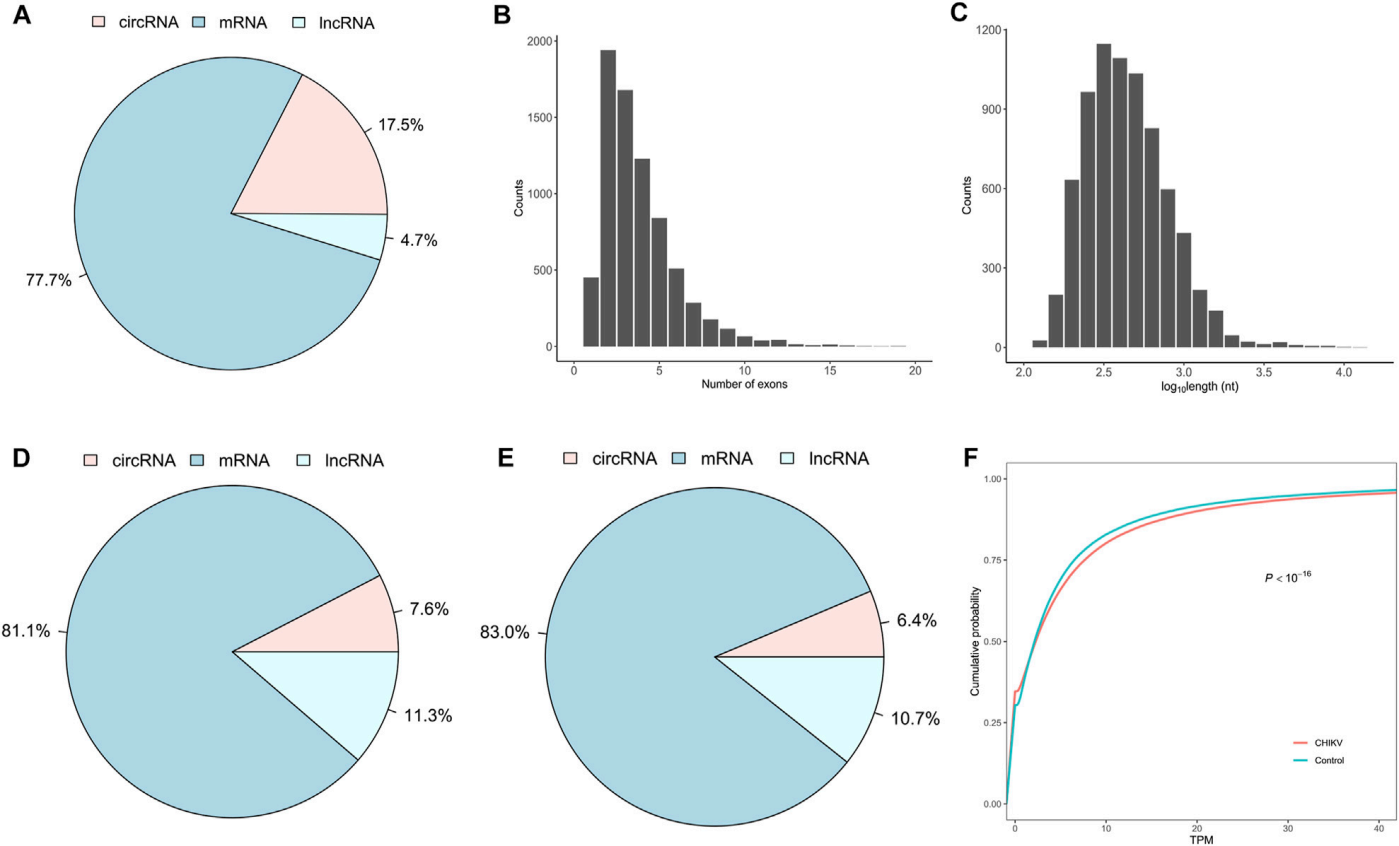
The expression landscape of peripheral whole blood transcriptome in the discovery set. **(A)** The fraction of expressed circRNA, mRNA and lncRNA species, **(B)** the distribution of exonic number and **(C)** the distribution of exonic length of exonic circRNAs within the blood transcriptome repertoire were plotted. Furthermore, the fraction of expression abundances of circRNAs, mRNAs and lncRNAs in patients with CHIKV infection **(D)** and healthy controls **(E)**, and the cumulative distribution of circRNA expressions in patients with CHIKV infection and healthy controls **(F)** were shown. The *P*-value of cumulative distribution comparison was obtained by *Kolmogorov-Smirnov* test.

To develop a circRNA signature that can differentiate CHIKV infectious patients from healthy controls, we identified and quantified the expression values of linear mRNAs, lncRNAs and circRNAs from RNA-seq data of whole blood samples of 39 patients with CHIKV infection and 20 healthy controls using our bioinformatics pipeline (see *Methods*). We observed that linear mRNAs had the highest number of expressed transcripts (77.7% of all expressed transcripts), while circRNAs had the lowest number of expressed transcripts (4.7% of all expressed transcripts) in all blood samples (Figure 2A). For the expressed circRNAs, most circRNAs had no more than five exons (Figure 2B) and were less than 1,000 base pairs at length (Figure 2C). In addition to the expressed number of transcripts, circRNAs also had the lowest abundance in the whole blood transcriptome in both CHIKV patients (Figure 2D) and healthy controls (Figure 2E). Similarly, linear mRNAs had the highest expressions in the transcriptome in all samples (Figures 2D,E). When comparing the circRNA expression abundance between patients with CHIKV infection and healthy controls, we observed significantly higher circRNA expressions in patients with CHIKV infection (*KS test*, *P-*value <10^–16^) (Figure 2F). In general, this *de novo* constructed human blood transcriptome repertoire had similar circRNA expression landscape to those we observed in previous studies (Wen and Gu, 2022; Qian et al., 2018).

### Identification of a blood circRNA signature in the discovery dataset

**FIGURE 3:**
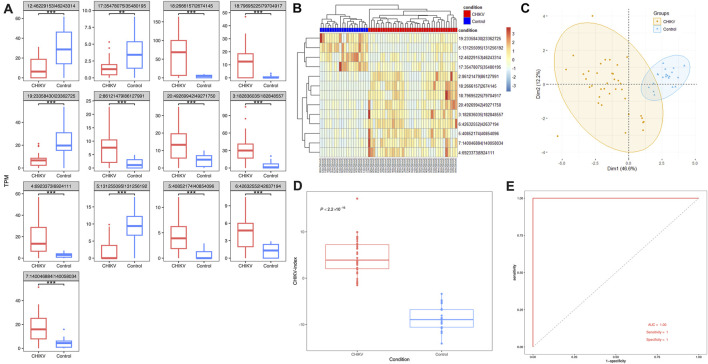
The performance of 13-circRNA signature in the discovery dataset. **(A)** The expression level of 13 biomarker circRNAs in patients with CHIKV infection and healthy controls and their differences between these two groups (*t*-test, *: *P* ≤ 0.05; **: *P* ≤ 0.01; ***: *P* ≤ 0.001). **(B)** The unsupervised hierarchical clustering and **(C)** principal component analysis was performed on 13 biomarker circRNA expression values of 43 blood samples at the acute phase of CHIKV infection and 43 blood samples at the convalescent phase of CHIKV infection. **(D)** Comparison of the CHIKV-index between patients with CHIKV infection at the acute phase and the convalescent phase was shown. The *P*-value was computed by *t*-test. **(E)** The ROC curve of the CHIKV-index based classification of patients with CHIKV infection and healthy controls.

Using a fitted linear model with controlling potential cofactors of age and gender, we screened out 13 differentially expressed circRNAs from the discovery dataset. Among them, 9 circRNAs were significantly upregulated and 4 circRNAs were significantly downregulated in patients with CHIKV infection (*t*-test: *P*-value <0.01) (Figure 3A; Supplementary Table S2). To evaluate the potential of using these 13 circRNAs as the diagnostic biomarkers of CHIKV infection, we performed unsupervised hierarchical clustering analysis and PCA analysis based on their expression values, respectively. Both cluster analysis (Figure 3B) and PCA analysis (Figure 3C) illustrated patients with CHIKV infection had totally distinct circRNA expression patterns from those of healthy controls. To further investigate the expression patterns of the parental genes of these 13 circRNAs, we analyzed their expression levels in the discovery dataset. The results revealed that 6 parental genes were significantly upregulated (*t*-test: *P*-value <0.05), two parental genes were significantly downregulated (*t*-test: *P*-value <0.01), and 4 parental genes showed no significant differences in expression (Supplementary Figure S1A). These findings suggest that while the parental genes exhibit some significant expression differences between CHIKV and healthy controls, the circRNAs show more pronounced changes, making them the primary contributors to the diagnostic capability of the 13-circRNA signature. Furthermore, we calculated the CHIKV-index of each sample using the expression values of these 13 circRNAs. We found that patients with CHIKV infection had significantly higher CHIKV-index than those of healthy controls (*t*-test, *P*-value <0.001) (Figure 3D). Finally, we performed a predictive analysis of CHIKV infection using the CHIKV-index, and observed an AUC value at 1, with a sensitivity of one and a specificity of 1. (Figure 3E). This suggested an excellent diagnostic performance of this 13-circRNA based signature in predicting CHIKV infection in the discovery cohort.

### Performance of the circRNA signature in the validation dataset

**FIGURE 4:**
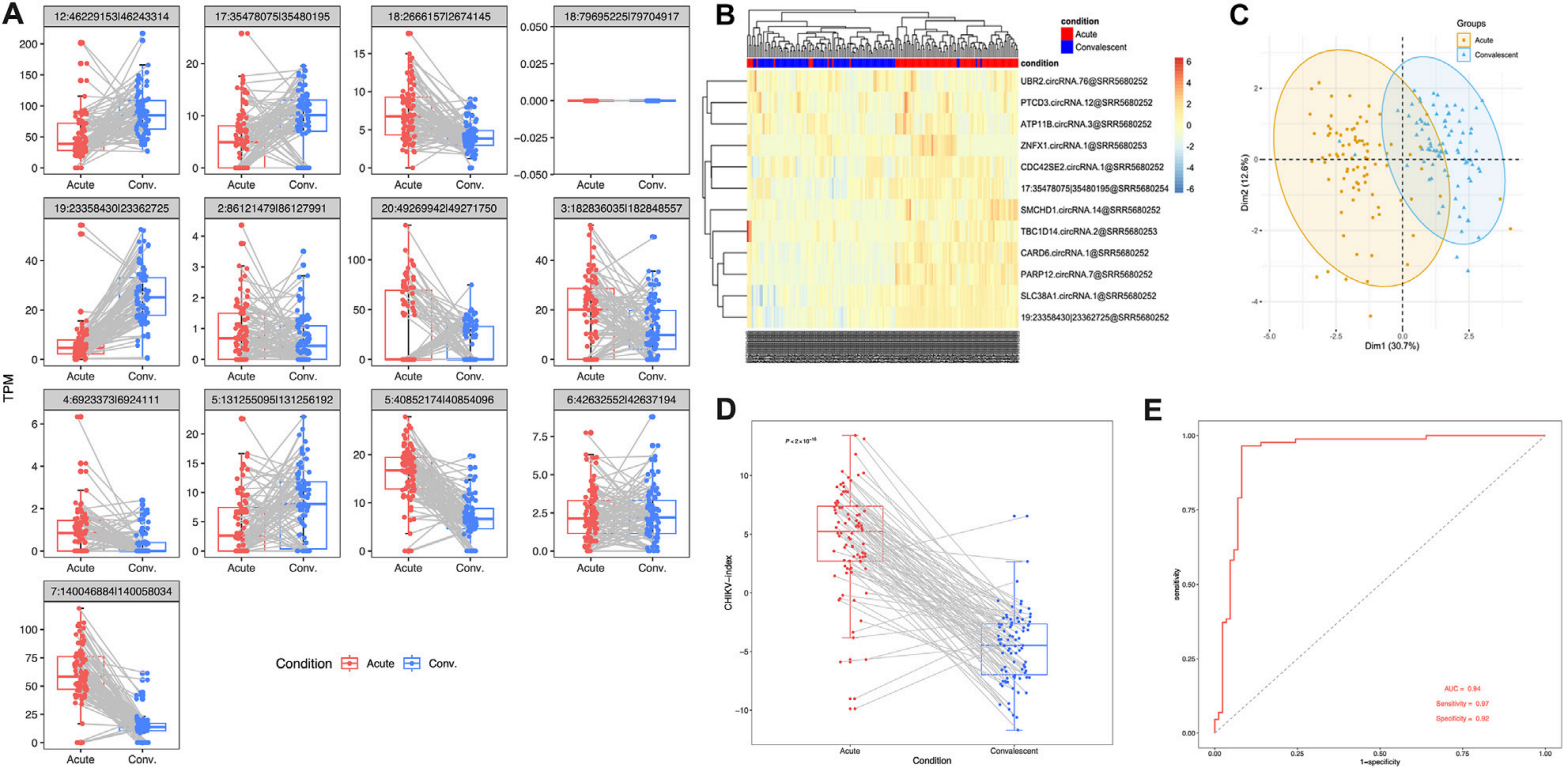
The performance of 13-circRNA signature in the validation dataset. **(A)** The expression values of 13 biomarker circRNAs in patients with acute CHIKV infection and those at the convalescent phase and the comparison between these two groups (paired *t*-test, *: *P* ≤ 0.05; **: *P* ≤ 0.01; ***: *P* ≤ 0.001, NS: non-significance). **(B)** The unsupervised hierarchical clustering analysis and **(C)** principal component analysis was performed on 13 circRNA expression values of blood samples in 39 patients with CHIKV infections and 20 healthy controls. **(D)** Comparison of CHIKV-index of circRNA signatures between acute phase and convalescent phase was shown. The *P*-value was computed by paired *t*-test. **(E)** The ROC curve of the CHIKV-index based classification of patients with CHIKV infection at the acute phase and those at the convalescent phase.

In order to validate the effectiveness of this 13-circRNA signature in CHIKV diagnosis, we measured the expression values of these circRNAs and assessed the diagnostic performance of this circRNA signature in an independent validation cohort. This validation cohort includes 43 children with CHIKV infection. Whole blood samples were collected at the acute infection phase and the convalescent phase, respectively, for each patient. Paired comparison showed that the expression level of most circRNA biomarkers remained significantly different between the acute infection phase and the convalescent phase, except for three circular transcripts (paired *t*-test, *P*-value <0.01; Figure 4A). Meanwhile, the trend of up- or downregulation was in accordance with that in the discovery cohort (Figure 4A). Both unsupervised hierarchical clustering analysis (Figure 4B) and PCA analysis (Figure 4C) on the expression values of circRNA biomarkers showed distinct expression patterns in blood samples of the acute phase and the convalescent phase of CHIKV infection. Similarly, in the validation cohort, we examined the expression levels of the parental genes of these circRNAs during the acute infection and convalescent phases. The analysis revealed that none of the 13 parental genes showed significant differential expression between the two phases (Supplementary Figure S1B). These findings highlight that while the parental genes exhibit stable expression patterns, the circRNAs themselves remain the key contributors to the diagnostic capability of the 13-circRNA signature. Furthermore, the CHIKV-index was significantly higher in blood samples of CHIKV infection at the acute phase than those at the convalescent phase (paired *t-test*: *P*-value <0.001, Figure 4D). The diagnostic power of CHIKV-index in differentiating CHIKV infections between acute phase and convalescent phase had an AUC value at 0.94, with a sensitivity of 0.97 and a specificity of 0.92. (Figure 4E). These results were consistent with those we observed in the discovery dataset (Figure 3), which confirmed the superior performance of our 13-circRNA signature in diagnosing patients with CHIKV infection.

### Association of circRNA signatures with disease phenotype

**FIGURE 5:**
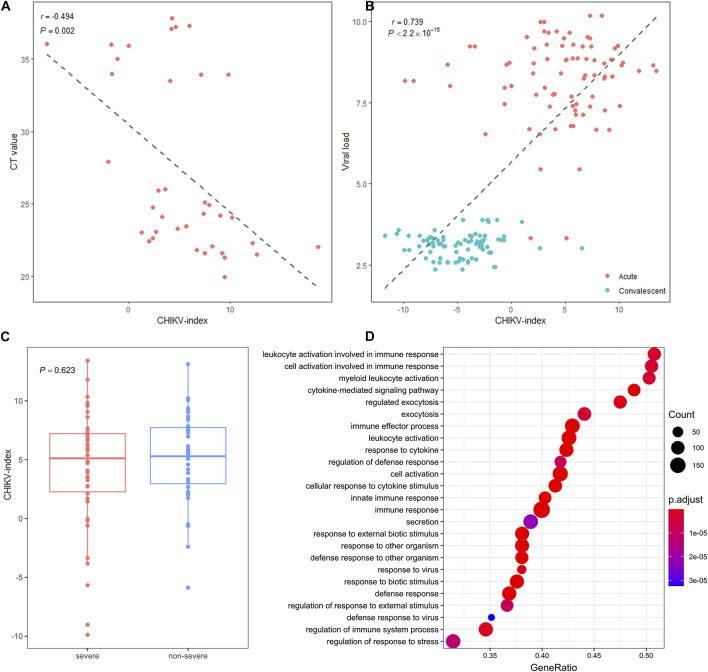
The association of circRNA signature-based score with disease phenotype. **(A)** The correlations of CHIKV-index with the CT values of CHIKV nucleotide by RT-qPCR detection in the discovery cohort and **(B)** the viral load in the validation cohort were shown. The *r* and *P*-value were computed by *Pearson* correlation test between the *x*- and *y*-axes. **(C)** The distribution and comparison of CHIKV-index values between severe and non-severe patients with CHIKV infection at the acute phase (*t*-test, NS: non-significance) was plotted. **(D)** The enriched dysregulated biological pathways of blood circRNAs in patients with CHIKV infection (GSEA analysis, *P*-value <0.05).

Next, we investigated clinical factors that may be related to this blood circRNA signature. We calculated the correlation between the CHIKV-index and two disease phenotypes, including viral load and disease severity, for each individual in both datasets. We observed a significantly negative correlation (*Pearson*’s correlation: *r* = −0.494, *P*-value = 0.002) between the CHIKV-index and the CT value of CHIKV qRT-PCR detection in the discovery cohort (Figure 5A). This correlation was also found between the CHIKV-index and the viral load in pediatric patients at the acute phase and convalescent phase of CHIKV infection in the validation cohort (*Pearson’*s correlation: *r* = 0.739, *P*-value <2.2*10^–16^) (Figure 5B). However, we did not see any statistical difference (*t*-test, *P*-value >0.05) between the CHIKV-index of patients with severe and non-severe infections at the acute phase (Figure 5C). This suggested that viral load, rather than the severity of CHIKV infection, may explain the variations of CHIKV-index among patients.

### Functional annotation of the dysregulated blood circRNAs upon CHIKV infection

To understand the functional roles that blood circRNAs made upon CHIKV infection, we first investigated the molecular function of the host gene of all 13 biomarker circRNAs (Supplementary Table S2). The parental genes of these biomarker circRNAs can be broadly categorized into three groups, including interferon-related genes, epigenetic-related genes and signal-transduction related genes. Next, we performed the GSEA analysis on expressed blood circRNAs in patients with CHIKV infection and healthy controls in the discovery cohort. We observed that the enriched biological pathways of dysregulated circRNAs were mainly involved in myeloid leukocyte activation, cytokine-mediated signaling pathway and exocytosis regulation (GSEA analysis, *P*-value <0.05) (Figure 5D). Therefore, these biomarker circRNAs may undertake immunoregulatory function in the activation of myeloid leukocytes and the regulation of antiviral responses against CHIKV invasion.

## Discussion

The worldwide outbreak of COVID-19 virus has made us realize the importance of rapid and accurate diagnostic methods for disease management and control (Pascarella et al., 2020). For CHIKV infection, it has dramatically expanded its geographic distribution to more than 100 countries and caused severe pubic concerns (Mourad et al., 2022). Therefore, there are urgent needs to develop a rapid and accurate diagnostic method for CHIKV infection. Current laboratory tests rely on serologic tests or molecular tests to detect the infectious status of CHIKV infection (Hakim and Aman, 2022). However, these tests detect the presence of the virus or CHIKV-specific antibodies, which may only be detected several days after infection (Mourad et al., 2022; Hakim and Aman, 2022). This made these tests not ideal tools for the prevention of CHIKV dissemination (Vairo et al., 2019; Mourad et al., 2022). To overcome the limitation of these methods, several recent studies have investigated the diagnostic power of serum cytokines (Krishnan et al., 2021) or neuroinflammatory biomarkers (Puccioni-Sohler et al., 2021) in cerebrospinal fluid for the diagnosis of CHIKV. In comparison to these existing diagnostic methods, gene expression in PBMCs or whole blood had the potential to assess the immune status of the body, to indicate the host response to pathogens and to serve as the early diagnostic markers for human diseases (Chaussabel, 2015). The reason why blood gene expression had the advantage of early diagnosis of infection is that PBMC or whole blood mainly consists immune cells, such as lymphocytes, monocytes and dendritic cells. These circulating immune cells are important components of the innate and adaptive immune system, which play an important role in the defense against exogenous organism invasion. As a successful example, Sutherland *et al.* developed a molecular biomarker test that profiled a panel of 42-gene expression markers from whole blood to differentiate sepsis patients from other patients with systemic inflammation due to physical trauma or wound healing (Sutherland et al., 2011). This biomarker test was further optimized and validated (Miller et al., 2018) to an FDA-approved molecular test, *SeptiCyte*, for the early diagnosis of sepsis. Although mRNA gene expressions can provide a more real-time and dynamic reflection to the immune status of the body, mRNAs are sensitive to sample processing and easy to introduce technical bias (Dvinge et al., 2014). Comparatively, circRNAs are more stable due to their unique circular structures that make circRNAs resistant to de-adenylation, decapping and exonucleases (Chen, 2016). Specifically, the median half-life of circRNAs was at least 2.5 times longer than that of their linear mRNA counterparts in mammary cells (Wen and Gu, 2022). Moreover, blood circRNAs have smaller transcriptome changes than mRNAs and lncRNAs upon sample processing delays (Wen and Gu, 2022). Thus, PBMC or whole blood circRNAs are promising disease biomarkers for the early diagnosis of CHIKV infection (Wen et al., 2021a).

Given these considerations, we tried to investigate the potential of using blood circRNAs as the diagnostic biomarkers of CHIKV infection. In our results, we observed abundant (Figures 2D,E) and somewhat significantly different circRNA expressions (Figures 3A, 4A) in the blood samples of both healthy controls and patients with CHIKV infections. Accordingly, we developed a panel of 13 blood circRNAs that could clearly distinguish CHIKV infectious patients from healthy controls with an AUC value at 1.000 (*P*-value <0.001) in the discovery cohort (Figure 3). In the validation cohort, this 13-circRNA signature could differentiate patients with acute CHIKV infection from those at the convalescent phase with an AUC value at 0.940 (*P*-value <0.001) (Figure 4). The perfect AUC value of 1.000 in the discovery cohort highlights the distinct circRNA expression patterns between CHIKV patients and healthy controls, emphasizing the diagnostic potential of the 13-circRNA signature. However, the relatively small sample size in the discovery cohort (39 CHIKV patients and 20 healthy controls) may increase the risk of overfitting and limit the representativeness of the results. Additionally, the validation cohort showed a slightly lower AUC value of 0.940, reflecting consistent diagnostic performance across independent datasets. While these results support the robustness of the 13-circRNA signature, further evaluations using larger and more diverse cohorts are essential to establish its generalizability and reliability in broader clinical applications. Additionally, we found a strong correlation of 13-circRNA expression based CHIKV-index with patient’s virus load in the discovery cohort (Figure 5A). Even though some patients had CHIKV nucleotide-based CT values higher than 35, our 13-circRNA signature based CHIKV-index can identify these patients from the healthy controls (Figure 3D). This suggests that this diagnostic signature we developed had the potential to identify patients with CHIKV infection before the viral load are high enough to be amplified by qRT-PCR. Similarly, we observed a significant decrease of CHIKV-index for CHIKV infectious patients at the convalescent phase in the validation cohort (Figure 5B). This also confirms the diagnostic value of the 13-circRNA signature and its correlation to viral load of CHIKV infection. However, we did not observe any significant changes of CHIKV-index for CHIKV infectious patients with different severity level (Figure 5C). This indicates that our 13-circRNA signature can be used as a diagnostic biomarker, rather than a prognostic biomarker, which had the potential for early diagnosis of CHIKV infection.

In our results, we observed that the dysregulation of blood circRNAs in patients with CHIKV infection is involved in myeloid leukocyte activation, exocytosis regulation and cytokine-mediated signaling pathway (Figure 5D). This suggests blood circRNAs may participate the host’s antiviral responses against CHIKV invasion via the activation of myeloid leukocytes. This is consistent to existing knowledge that CHIKV mainly affects human macrophages and monocytes (Bishop et al., 2022), which may induce the host’s response to its infection. Moreover, those blood biomarker circRNAs were largely derived from three functional gene groups (Supplementary Table S2). First, ZNFX1 and PARP12 are both interferon-stimulated genes, which can enhance the host’s immune response to virus. Second, SMCHD1 and UBR2 are genes related to epigenetic regulation. Specifically, SMCHD1 is associated with DNA methylation, which was identified as a pivotal cellular factor that restricts Kaposi’s sarcoma-associated herpesvirus lytic reactivation. UBR2 is predicted to be part of ubiquitin ligase complex, which was involved in the negative regulation of TOR signaling. Third, SLC38A1, CDC42SE2 and CARD6 are signal transduction-related genes, which were likely to perform some regulatory functions via signal transduction in immune responses. The functional annotation of these blood circRNAs confirms their implication in host’s response to CHIKV infection.

Although we suggested circRNAs’ utility as CHIKV diagnosis biomarkers, further analysis was conducted to explore their specificity against other infectious diseases. Using data from the *PRJNA588242* dataset, which includes samples from tuberculosis (TB) patients and healthy controls, we analyzed the expression of five circRNAs (due to data limitations) (Tornheim et al., 2020). The results showed no significant differences between the TB and healthy control groups (Supplementary Figure S2A). Furthermore, in the *PRJNA901461* dataset, which includes samples from *COVID-19* patients and healthy controls, we analyzed the expression of five circRNAs (Wang et al., 2022). Of these, three circRNAs showed no significant differences between the two groups, while two circRNAs were significantly differentially expressed (*t*-test, *P* ≤ 0.001) (Supplementary Figure S2B). These findings suggest that the 13-circRNA signature may have differential potential for CHIKV infection, but further validation across broader datasets and infectious diseases is essential to conclusively establish its diagnostic specificity. In future studies, it is important to address several limitations of this research. First, the sample size of both the discovery and validation datasets remains relatively small, which may affect the statistical robustness and generalizability of the findings. Second, dataset heterogeneity, including differences in sample composition, experimental conditions, and patient demographics, could influence the results’ consistency and reliability. This requires a large prospective cohort to validate the diagnostic power of our 13-circRNA signature. Finally, several other diseases, such as *Dengue* virus and *Zika* virus infections (de St Maurice et al., 2021), should be included in future cohorts to enhance the specificity of screened diagnostic biomarkers.

The 13-circRNA signature demonstrates promise as a non-invasive and accessible diagnostic tool for CHIKV infection. Unlike *qRT-PCR*, which requires sufficient viral load for detection, circRNAs can be reliably quantified even in the early stages of infection, providing a clear advantage for timely diagnosis. Furthermore, the intrinsic stability of circRNAs may make them suitable for field diagnostics in resource-limited settings, as they are less sensitive to sample handling and storage conditions compared to mRNA-based tests. However, several challenges remain in harnessing circRNA as diagnostic biomarkers. Firstly, while the 13-circRNA signature showed promising diagnostic performance, its specificity against other infectious diseases such as *Dengue* and *Zika* virus infections has yet to be comprehensively assessed. Additionally, standardized protocols for circRNA detection are crucial for ensuring reproducibility, but the complexity of analytical pipelines and the reliance on advanced technologies may hinder clinical implementation. Cost and accessibility remain significant barriers, especially in resource-limited regions. Addressing these challenges will be essential for translating circRNA-based diagnostics into practical tools for disease management. In conclusion, we suggested that blood circRNAs are potentially reliable biomarkers to diagnose CHIKV infection in its early phase, which may have translational implications in the management of CHIKV infection.

## Data Availability

Existing datasets are available in a publicly accessible repository: Publicly available datasets were analyzed in this study. This data can be found here: [https://www.ncbi.nlm.nih.gov/bioproject/390289/PRJNA390289; https://www.ncbi.nlm.nih.gov/bioproject/PRJNA507472/PRJNA507472].

## References

[B1] Awan F. M. Yang B. B. Naz A. Hanif A. Ikram A. Obaid A. (2021). The emerging role and significance of circular RNAs in viral infections and antiviral immune responses: possible implication as theranostic agents. RNA Biol. 18 (1), 1–15. 10.1080/15476286.2020.1790198 PMC7833768 32615049

[B2] Bishop C. R. Caten F. T. Nakaya H. I. Suhrbier A. (2022). Chikungunya patient transcriptional signatures faithfully recapitulated in a C57BL/6J mouse model. Front. Immunol. 13, 1092370. 10.3389/fimmu.2022.1092370 36578476 PMC9791225

[B3] Chaussabel D. (2015). Assessment of immune status using blood transcriptomics and potential implications for global health. Seminars Immunol. 27, 58–66. 10.1016/j.smim.2015.03.002 25823891

[B4] Chen L.-L. (2016). The biogenesis and emerging roles of circular RNAs. Nat. Rev. Mol. Cell Biol. 17 (4), 205–211. 10.1038/nrm.2015.32 26908011

[B5] Chen L.-L. (2020). The expanding regulatory mechanisms and cellular functions of circular RNAs. Nat. Rev. Mol. Cell Biol. 21 (8), 475–490. 10.1038/s41580-020-0243-y 32366901

[B6] de St Maurice A. Ervin E. Chu A. (2021). Ebola, Dengue, chikungunya, and Zika infections in neonates and infants. Clin. Perinatol. 48 (2), 311–329. 10.1016/j.clp.2021.03.006 34030816

[B7] Dvinge H. Ries R. E. Ilagan J. O. Stirewalt D. L. Meshinchi S. Bradley R. A.-O. (2014). Sample processing obscures cancer-specific alterations in leukemic transcriptomes. PNAS 111, 16802–16807. 10.1073/pnas.1413374111 25385641 PMC4250124

[B8] George M. Abraham M. MPH, FACP, FIDSA (2019). Chikungunya virus: a global threat and menace. Infect. Dis. Clin. Pract. 27 (2), 67. 10.1097/ipc.0000000000000706

[B9] Gu W. Shi J. Liu H. Zhang X. Zhou J. J. Li M. (2020). Peripheral blood non-canonical small non-coding RNAs as novel biomarkers in lung cancer. Mol. Cancer 19 (1), 159. 10.1186/s12943-020-01280-9 33176804 PMC7659116

[B10] Hakim M. S. Aman A. T. (2022). Understanding the biology and immune pathogenesis of chikungunya virus infection for diagnostic and vaccine development. Viruses 15 (1), 48. 10.3390/v15010048 36680088 PMC9863735

[B11] Huits R. De Kort J. Van Den Berg R. Chong L. Tsoumanis A. Eggermont K. (2018). Chikungunya virus infection in Aruba: diagnosis, clinical features and predictors of post-chikungunya chronic polyarthralgia. PLoS One 13 (4), e0196630. 10.1371/journal.pone.0196630 29709007 PMC5927412

[B12] Krishnan S. M. Mahalingam J. Sabarimurugan S. Muthu T. Venkidasamy B. Krishnasamy K. (2021). Comparison of cytokine expression profile in chikungunya and Dengue Co-infected and mono-infected patients' samples. Pathogens 10 (2), 166. 10.3390/pathogens10020166 33557110 PMC7913810

[B13] Michlmayr D. Pak T. R. Rahman A. H. Amir E. D. Kim E. Y. Kim-Schulze S. (2018). Comprehensive innate immune profiling of chikungunya virus infection in pediatric cases. Mol. Syst. Biol. 14 (8), e7862. 10.15252/msb.20177862 30150281 PMC6110311

[B14] Miller R. R. 3rd Lopansri B. K. Burke J. P. Levy M. Opal S. Rothman R. E. (2018). Validation of a host response assay, SeptiCyte LAB, for discriminating sepsis from systemic inflammatory response syndrome in the ICU. Am. J. Respir. Crit. Care Med. 198 (7), 903–913. 10.1164/rccm.201712-2472OC 29624409 PMC6835074

[B15] Min J. Li Y. Li X. Wang M. Li H. Bi Y. (2023). The circRNA circVAMP3 restricts influenza A virus replication by interfering with NP and NS1 proteins. PLoS Pathog. 19 (8), e1011577. 10.1371/journal.ppat.1011577 37603540 PMC10441791

[B16] Mounce B. C. Cesaro T. Vlajnić L. Vidiņa A. Vallet T. Weger-Lucarelli J. (2017). Chikungunya virus overcomes polyamine depletion by mutation of nsP1 and the opal stop codon to confer enhanced replication and fitness. J. Virol. 91 (15). 10.1128/JVI.00344-17 PMC5512238 28539441

[B17] Mourad O. Makhani L. Chen L. H. (2022). Chikungunya: an emerging public health concern. Curr. Infect. Dis. Rep. 24 (12), 217–228. 10.1007/s11908-022-00789-y 36415286 PMC9672624

[B18] Pascarella G. Strumia A. Piliego C. Bruno F. Del Buono R. Costa F. (2020). COVID-19 diagnosis and management: a comprehensive review. J. Intern Med. 288 (2), 192–206. 10.1111/joim.13091 32348588 PMC7267177

[B19] Puccioni-Sohler M. da Silva S. J. Faria L. C. S. Cabral D. Cabral-Castro M. J. (2021). Neopterin and CXCL-10 in cerebrospinal fluid as potential biomarkers of neuroinvasive Dengue and chikungunya. Pathogens 10 (12), 1626. 10.3390/pathogens10121626 34959581 PMC8706264

[B20] Qian Z. Liu H. Li M. Shi J. Li N. Zhang Y. (2018). Potential diagnostic power of blood circular RNA expression in active pulmonary tuberculosis. EBioMedicine 27, 18–26. 10.1016/j.ebiom.2017.12.007 29248507 PMC5828303

[B21] Soares-Schanoski A. Baptista Cruz N. de Castro-Jorge L. A. de Carvalho R. V. H. Santos C. A. D. Ros N. D. (2019). Systems analysis of subjects acutely infected with the Chikungunya virus. PLoS Pathog. 15 (6), e1007880. 10.1371/journal.ppat.1007880 31211814 PMC6599120

[B22] Soneson C. Love M. I. Robinson M. D. (2015). Differential analyses for RNA-seq: transcript-level estimates improve gene-level inferences. F1000Res 4, 1521. 10.12688/f1000research.7563.2 26925227 PMC4712774

[B23] Subramanian A. Tamayo P. Fau - Mootha V. K. Mootha Vk Fau - Mukherjee S. Mukherjee S. Fau - Ebert B. L. (2005). Gene set enrichment analysis: a knowledge-based approach for interpreting genome-wide expression profiles. PNAS 102 (43), 15545–15550. 10.1073/pnas.0506580102 16199517 PMC1239896

[B24] Sutherland A. Thomas M. Brandon R. A. Brandon R. B. Lipman J. Tang B. (2011). Development and validation of a novel molecular biomarker diagnostic test for the early detection of sepsis. Crit. Care 15 (3), R149. 10.1186/cc10274 21682927 PMC3219023

[B25] Suzuki Y. (2023). Interferon-induced restriction of Chikungunya virus infection. Antivir. Res. 210, 105487. 10.1016/j.antiviral.2022.105487 36657882

[B26] Tornheim J. A. Madugundu A. K. Paradkar M. Fukutani K. F. Queiroz A. T. L. Gupte N. (2020). Transcriptomic profiles of confirmed pediatric tuberculosis patients and household contacts identifies active tuberculosis, infection, and treatment response among Indian children. J. Infect. Dis. 221 (10), 1647–1658. 10.1093/infdis/jiz639 31796955 PMC7184902

[B27] Vairo F. Haider N. Kock R. Ntoumi F. Ippolito G. Zumla A. (2019). Chikungunya: epidemiology, pathogenesis, clinical features, management, and prevention. Infect. Dis. Clin. North Am. 33 (4), 1003–1025. 10.1016/j.idc.2019.08.006 31668189

[B28] Wang Y. Schughart K. Pelaia T. M. Chew T. Kim K. Karvunidis T. (2022). Blood transcriptome responses in patients correlate with severity of COVID-19 disease. Front. Immunol. 13, 1043219. 10.3389/fimmu.2022.1043219 36741372 PMC9896980

[B29] Wen G. Gu W. (2022). Circular RNAs in peripheral blood mononuclear cells are more stable than linear RNAs upon sample processing delay. J. Cell Mol. Med. 26 (19), 5021–5032. 10.1111/jcmm.17525 36039821 PMC9549506

[B30] Wen G. Li M. Li F. Yang Z. Zhou T. Gu W. (2021b). AQUARIUM: accurate quantification of circular isoforms using model-based strategy. Bioinformatics 37 (24), 4879–4881. 10.1093/bioinformatics/btab435 34115093

[B31] Wen G. Zhou T. Gu W. (2021a). The potential of using blood circular RNA as liquid biopsy biomarker for human diseases. Protein Cell 12 (12), 911–946. 10.1007/s13238-020-00799-3 33131025 PMC8674396

[B32] Xin R. Gao Y. Gao Y. Wang R. Kadash-Edmondson K. E. Liu B. (2021). isoCirc catalogs full-length circular RNA isoforms in human transcriptomes. Nat. Commun. 12 (1), 266. 10.1038/s41467-020-20459-8 33436621 PMC7803736

[B33] Yan L. Chen Y. G. (2020). Circular RNAs in immune response and viral infection. Trends Biochem. Sci. 45 (12), 1022–1034. 10.1016/j.tibs.2020.08.006 32900574 PMC7642119

[B34] Yao W. Pan J. Liu Z. Dong Z. Liang M. Xia S. (2021). The cellular and viral circRNAome induced by respiratory syncytial virus infection. mBio 12 (6), e0307521. 10.1128/mBio.03075-21 34872355 PMC8649777

[B35] Yap G. Pok K. Y. Lai Y. L. Hapuarachchi H. C. Chow A. Leo Y. S. (2010). Evaluation of Chikungunya diagnostic assays: differences in sensitivity of serology assays in two independent outbreaks. PLoS Negl. Trop. Dis. 4 (7), e753. 10.1371/journal.pntd.0000753 20651930 PMC2907414

[B36] Yu G. Wang Lg Fau - Han Y. Han Y. Fau - He Q.-Y. He Q. Y. (2012). clusterProfiler: an R package for comparing biological themes among gene clusters, 1557–8100. (Electronic)). 10.1089/omi.2011.0118 PMC3339379 22455463

[B37] Yuan D. Ahamed A. Burgin J. Cummins C. Devraj R. Gueye K. (2023). The European nucleotide archive in 2023. Nucleic Acids Res. 52, D92–D97. 10.1093/nar/gkad1067 PMC10767888 37956313

[B38] Zhang R. Kim A. S. Fox J. M. Nair S. Basore K. Klimstra W. B. (2018). Mxra8 is a receptor for multiple arthritogenic alphaviruses. Nature 557 (7706), 570–574. 10.1038/s41586-018-0121-3 29769725 PMC5970976

[B39] Zheng Y. Ji P. Chen S. Hou L. Zhao F. (2019). Reconstruction of full-length circular RNAs enables isoform-level quantification. Genome Med. 11 (1), 2. 10.1186/s13073-019-0614-1 30660194 PMC6339429

[B40] Zhou T. Xie X. Li M. Shi J. Zhou J. J. Knox K. S. (2018). Rat BodyMap transcriptomes reveal unique circular RNA features across tissue types and developmental stages. RNA 24 (11), 1443–1456. 10.1261/rna.067132.118 30093490 PMC6191709

