# Supplementary material for: The identification of a blood circular RNA signature that differentiates Chikungunya virus infection
Source: Front Genet. 2025 Apr 29;16:1602177. doi: 10.3389/fgene.2025.1602177 (PMC12069458; doi:10.3389/fgene.2025.1602177)
Supplement: Supplementary file 1 [file DataSheet1.docx]

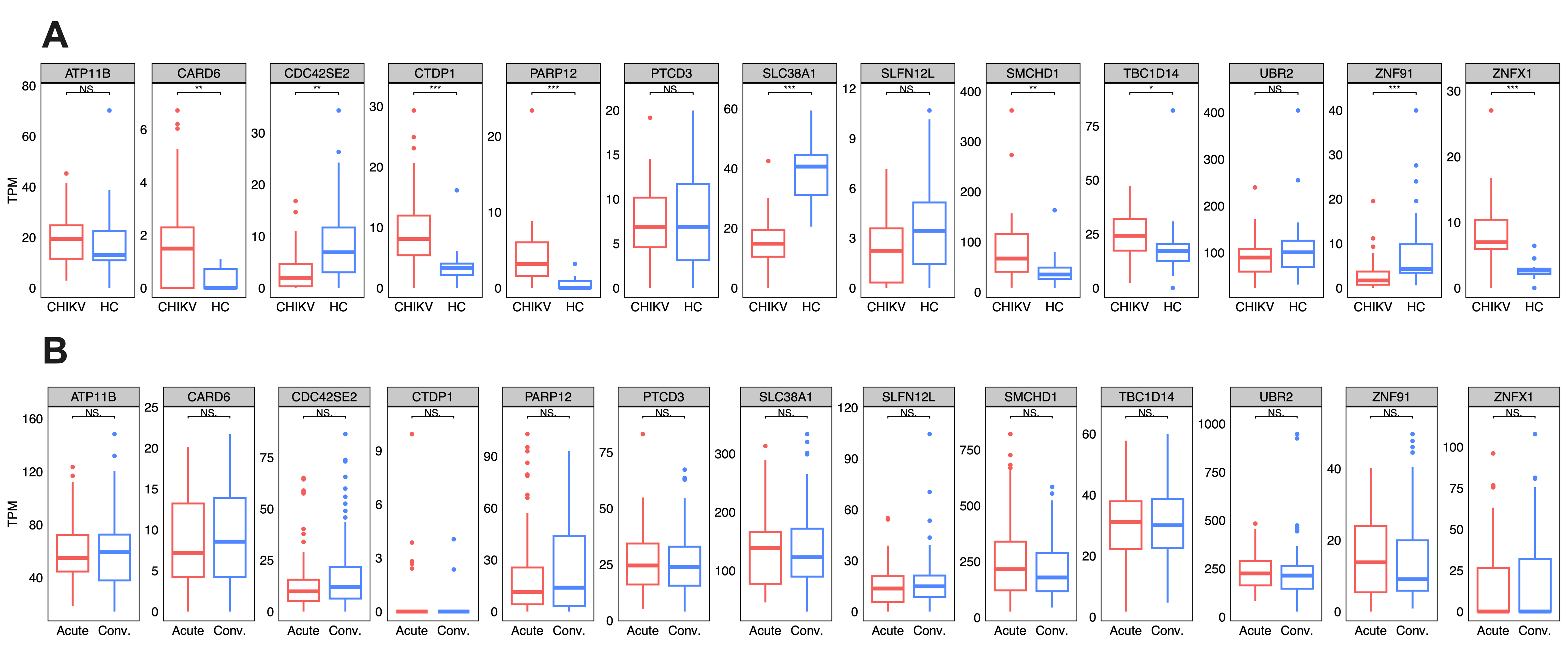


**Supplementary Figure S1. Expression levels of the parent genes of circRNAs in the 13-circRNA signature.** The expression levels of parent genes of 13 circRNAs in **(A)** the discovery cohort and **(B)** validation cohort were plotted. (*NS.*: Non-Significant; *: *P* ≤ 0.05; **: *P* ≤ 0.01; ***: *P* ≤ 0.001)**.**


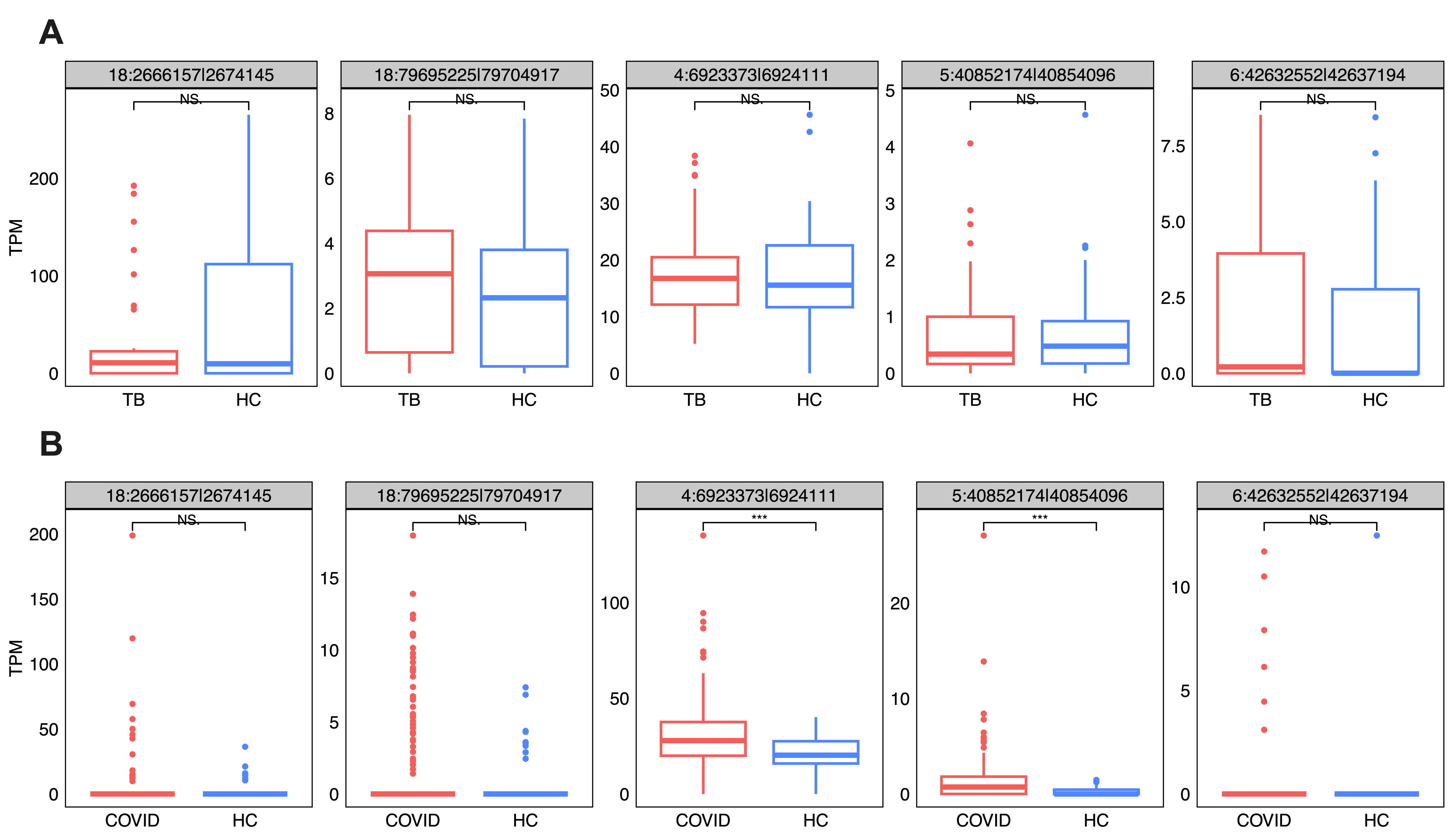


**Supplementary Figure S2. Expression profiles of circRNAs in the 13-circRNA signature in the dataset of additional infectious diseases.** The expression values of 13 circRNAs in the **(A)** TB and HC samples and **(B)** COVID-19 and HC samples. (TB: Tuberculosis; HC: Healthy Control; *NS.*: Non-Significant; *: *P* ≤ 0.05; **: *P* ≤ 0.01; ***: *P* ≤ 0.001).
